# A genome-wide screen for FTY720-sensitive mutants reveals genes required for ROS homeostasis

**DOI:** 10.15698/mic2017.12.601

**Published:** 2017-11-27

**Authors:** Kanako Hagihara, Kanako Kinoshita, Kouki Ishida, Shihomi Hojo, Yoshinori Kameoka, Ryosuke Satoh, Teruaki Takasaki, Reiko Sugiura

**Affiliations:** 1 Laboratory of Molecular Pharmacogenomics, Department of Pharmaceutical Sciences, Faculty of Pharmacy, Kindai University, 3-4-1 Kowakae, Higashi-Osaka City, Osaka 577-8502, Japan.

**Keywords:** FTY720, ROS, chemical genomics, fission yeast, mechanism of cell death

## Abstract

Fingolimod hydrochloride (FTY720), a sphingosine-1-phosphate (S1P) analogue, is an approved immune modulator for the treatment of multiple sclerosis (MS). Notably, in addition to its well-known mode of action as an S1P modulator, accumulating evidence suggests that FTY720 induces apoptosis in various cancer cells via reactive oxygen species (ROS) generation. Although the involvement of multiple signaling molecules, such as JNK (Jun N-terminal kinase), Akt (alpha serine/threonine-protein kinase) and Sphk has been reported, the exact mechanisms how FTY720 induces cell growth inhibition and the functional relationship between FTY720 and these signaling pathways remain elusive. Our previous reports using the fission yeast *Schizosaccharomyces pombe* as a model system to elucidate FTY720-mediated signaling pathways revealed that FTY720 induces an increase in intracellular Ca^2+^ concentrations and ROS generation, which resulted in the activation of the transcriptional responses downstream of Ca^2+^/calcineurin signaling and stress-activated MAPK signaling, respectively. Here, we performed a genome-wide screening for genes whose deletion induces FTY720-sensitive growth in *S. pombe* and identified 49 genes. These gene products are related to the biological processes involved in metabolic processes, transport, transcription, translation, chromatin organization, cytoskeleton organization and intracellular signal transduction. Notably, most of the FTY720-sensitive deletion cells exhibited NAC-remedial FTY720 sensitivities and dysregulated ROS homeostasis. Our results revealed a novel gene network involving ROS homeostasis and the possible mechanisms of the FTY720 toxicity.

## INTRODUCTION

Fingolimod (FTY720), a Sphingosine 1-phosphate (S1P) receptor modulator, is an immune modulator approved to treat multiple sclerosis 1,2. The mechanism of FTY720 action is primarily related to lymphocyte sequestration in lymphoid tissues. Notably, FTY720 has been reported to exert antitumor properties against breast cancers 3,4,5, glioblastoma 6,7,8, hepatocellular carcinoma 9,10,11, and malignant mesothelioma 12, implying that FTY720 action is involved in multiple intracellular signaling pathways related to cancer signaling. Intriguingly, FTY720 exerts its anticancer property largely independent of the phosphorylation of the drug, suggesting that activation of S1P receptor is not relevant for cell death 4,8,11,12,13,14. Various signaling molecules such as protein phosphatase 2A, JNK, Akt, and Sphk have been found to mediate anticancer effects associated with FTY720 4,8,12,15. In particular, the importance of the FTY720-mediated ROS generation in inducing apoptosis in various cancer cell types has been highlighted in several reports 8,10,11,14,15,16.

We have been using the fission yeast *Schizosaccharomyces pombe *as a powerful model system for studying the mechanism(s) of drug action and genetic contexts associated with drug sensitivity and/or resistance 17,18,19,20. These include chemical genomics studies which identify genes to determine the sensitivity/tolerance to FK506, valproic acid, and rapamycin 17,19,20. Furthermore, this organism is an excellent model for drug discovery, as we recently developed a drug screening system and have successfully discovered ACA-28, a novel ERK signaling modulator which preferentially kills human melanoma cells (ACA-28) 21.

We also extended our research to analyze the signaling pathways mediating FTY720-induced cell growth defects, as this compound may possess multiple biological functions other than serving as an S1P modulator as shown above. This multiple FTY720 activities could be achieved presumably by interacting with as-yet-unidentified targets and thereby modulating various signaling pathways. Our study showed that FTY720 stimulated ROS production and Ca^2+^ influx, followed by subsequent activation of the Sty1/Spc1 stress-activated MAPK signaling pathway and Ca^2+^/calcineurin signaling, respectively 22,23. Consistently, cells lacking the components of the stress-activated Sty1 MAPK signaling pathway exhibited growth retardation in media containing FTY720 with enhanced ROS production, indicating that an appropriate balance of ROS signaling plays a key role as a determinant of FTY720 toxicity. Intriguingly, the fission yeast genome does not express the S1P receptor, which, in combination with its powerful genetic tools and resources, would be beneficial to clearly delineate components and signaling pathways relevant to FTY720-mediated responses with a special focus on ROS.

Here, we performed a systematic screen of the set of ~4,000 viable *S. pombe* haploid gene deletion mutants and have identified 49 genes whose deletion causes hypersensitivity to FTY720. Clustering of the FTY720 sensitive genes on the basis of their cross-sensitivity to hydrogen peroxide (H_2_O_2_), a typical ROS inducer, revealed that more than 40 gene deletion cells exhibited FTY720-speficific sensitivity and 9 gene deletion cells showed cross-sensitivity. Notably, most of the FTY720-sensitive gene deletion cells exhibited NAC (N-acetylcysteine)-remedial growth defects as well as deregulated ROS accumulation. Of the 49 FTY720-sensitive genes, 37 are conserved from yeast to humans and include factors involved in signaling, transcription, chromatin organization, translation, metabolic processes, transport, cytoskeletal organization, and meiosis. Our systematic study provides valuable information on the complex signaling network mediated by FTY720 and ROS regulation. This paper will discuss possible mechanisms of the FTY720-regulated ROS homeostasis and FTY720-induced toxicity.

## RESULTS

### Genome-wide identification of genes associated with sensitivity to FTY720

We performed a genome-wide screen to identify non-essential genes whose deletion confers sensitivity to FTY720 using the BIONEER *S.* *pombe *haploid knockout strain collection (Version 4). Because the genotype of this strain collection is* h^+^ leu1-32 ura4-D18 ade6-M210/ade6-M216 *and the selection markers are *Kan*MX4 24, we used KP178 (*h^-^ leu1-32 ura4-D18 ade6-M210*) as a wild-type (wt) control strain. Cell cultures were grown in yeast extract plus supplements (YES).

**Figure 1:**
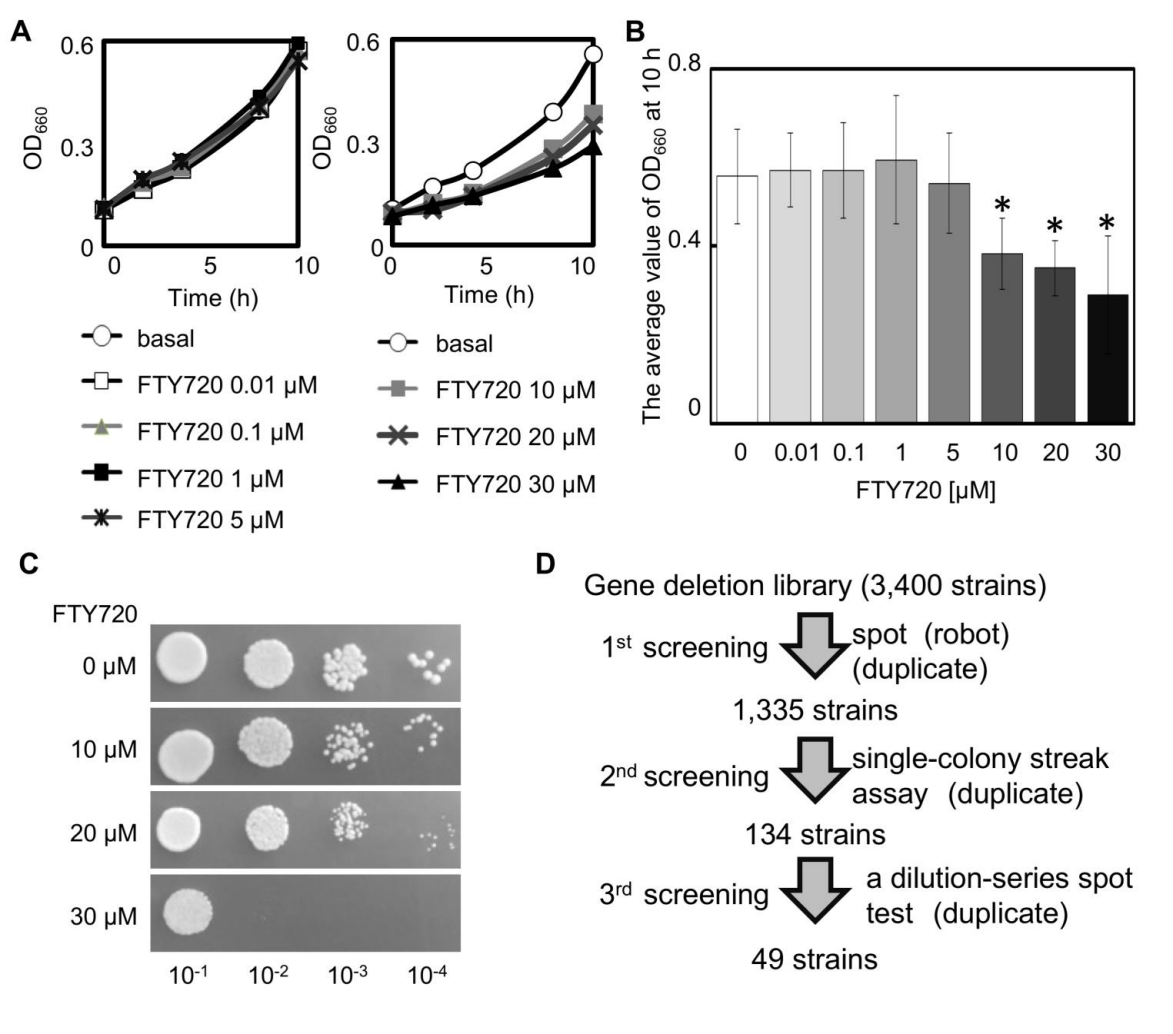
FIGURE 1: Fission yeast cells are sensitive to FTY720. ** (A)** Quantitative measurements of cell growth in the presence of FTY720. The wt strain (KP178) was grown in liquid YES cultures to an OD_660_ of 0.3 and was treated with FTY720 with the concentrations indicated, and the quantitative measurements of cell growth rates were performed using a microplate reader (SunriseTM series, Tecan, Switzerland). The growth curves of the cells presented are the average for three independent curves. ** (B)** The histograms show the OD_660_ at 10 h, as indicated in Figure 1A. The data were averaged from three independent experiments. Error bars, SD, *P < 0.05, significantly different from FTY720 0 µM using Williams’ test. **(C)** A serial dilution assay of the wt strain grown in YES medium or YES medium containing the indicated concentrations of FTY720. Cells were incubated for 4 days at 27°C. **(D)** Work-flow of the screening procedure to uncover genes required for FTY720-resistance. A total of 3,400 strains from Bioneer ver4.0 library were screened. Each of the strains was automatically spotted on YES media containing 0, 10, 20 µM FTY720 and serially diluted and manually streaked and subsequently spotted. 49 strains were found to be repeatedly showing sensitivity on FTY720.

The influence of the compound (FTY720) on the growth of the adenine-requiring wt cells (KP178) was evaluated in the YES liquid medium in order to assess the optimum concentrations to screen for FTY720 sensitive strains using the BIONEER collection (comprised of adenine requiring strains). The results showed that FTY720 induced growth inhibition of KP178 at concentrations ranging from 10 to 30 µM (Figure 1A, B). Furthermore, KP178 failed to grow in the presence of 30 µM FTY720 (Figure 1C). We therefore decided to use 10 and 20 µM FTY720 as concentrations to analyze the sensitivities of various deletion strains to FTY720 in comparison with that of the wt cells.

In the primary screening, each strain from the deletion library (comprised of 3,400 gene deletion strains) was incubated in a rich YES agar plate as a growth reference and then spotted onto agar plates containing 10 or 20 µM FTY720, assisted by a robot system (ROTOR, SINGER INSTRUMENT, UK)(Figure 1D). From this primary screen, 1,335 mutant strains that displayed varying degrees of growth defects were isolated in the media containing 10 or 20 µM FTY720. The 1,335 strains that had growth defects with 10 µM FTY720 were retested by single-colony streak of logarithmic phase cultures of each strain onto YES plates containing 0, 10, or 20 μM FTY720 in duplicate. Subsequently, 134 mutants were evaluated as sensitive, with varying degrees of sensitivity.

**Figure 2:**
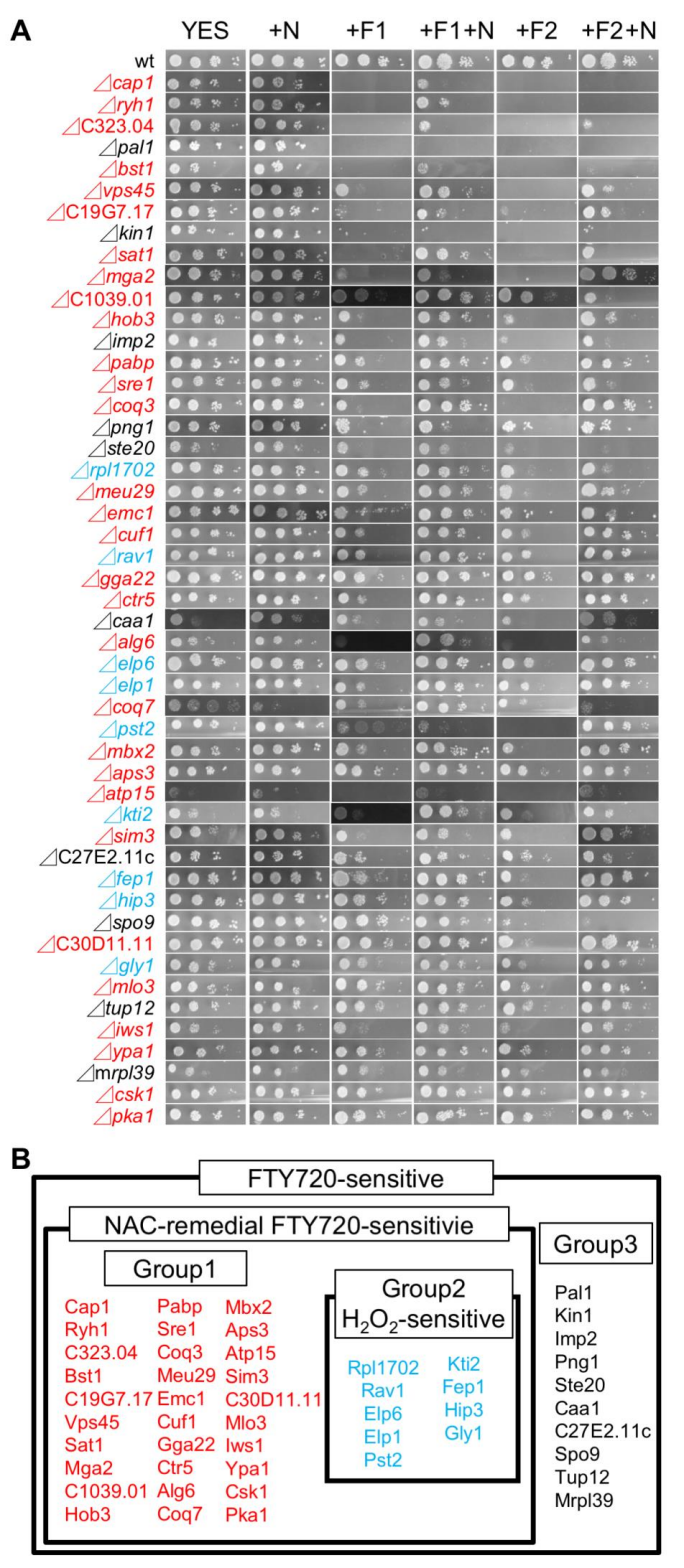
FIGURE 2: FTY720-sensitivity screening using dilution-series spot assay for the 49 KO strains identified in the third screen. ** (A)** Raw data of chemical genomics screening on the YES plate with 0, 10, 20 μM FTY720 (YES, +F10, +F20) in the absence/presence of 5 mM NAC (+N). The genes shown in red affected the cytotoxicity of FTY720 and the reversibility by NAC (Group1). The genes shown in blue had growth defects in H_2_O_2_ sensitivity (Group2). **(B)** Venn diagram showing the result of A.

In order to further retest for sensitivity and to determine the degree of sensitivity associated with 134 FTY720-sensitive mutants selected by the single-colony streak method as above, a dilution-series spot test of these mutants comparing the wt cells was performed (Figure 1D). Serial dilution of these 134 mutants (identified by single-colony streak assay) was spotted onto YES plates containing 0 (YES), 10 (+F10), or 20 μM FTY720 (+F20), and cultured at 27^°^C for 4 days (Figure 2A).

**Figure 3:**
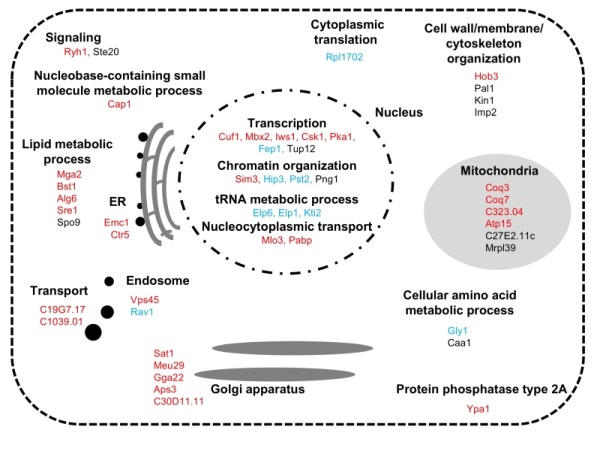
FIGURE 3: Summary of identified the FTY720-sensitive genes Functional classification and sub-cellular localization of FTY720 sensitive gene products.

Ultimately, 39 mutants were scored as severely sensitive (-3), and 10 mutants were scored as moderately sensitive (-2) (Table S1). The list of the FTY720-sensitive genes is shown in Table S1 along with the systemic name, the common name of the gene from *S. pombe* (if available), and a brief description of the function of each gene product is also indicated. As shown in Table S1, the biological functions of these gene products were classified into 8 categories including metabolic processes, transcription, chromatin organization, translation, transport, signaling, cytoskeletal organization, and others, which will be further described in Figure 3 and in the Discussion.

### Most of the FTY720-sensitive gene deletion cells exhibited NAC-remedial growth defects associated with FTY720 and/or H_2_O_2_ treatment

The addition of the ROS scavenger NAC (N-acetylcysteine) was shown to reverse the growth defects associated with FTY720 treatment by decreasing intracellular ROS levels 23. Therefore, it is assumed that if the addition of NAC rescued the FTY720-sensitivities associated with the 49 isolated FTY720-sensitive deletion strains, the mechanisms of the observed phenotypes in the relevant strains might involve deteriorated ROS homeostasis. Based on this assumption, we examined whether the addition of NAC can recover FTY720-sensitive phenotypes in the 49 mutant cells. Therefore, we performed spot tests and compared the FTY720 sensitivity in the presence of (+N, +F10+N, +F20+N) and the absence of (YES, +F10, +F20) 5 mM NAC (Figure 2A).

Notably, among the 49 mutants, 39 exhibited NAC-remedial FTY720-sensitivities, whereas 10 mutants were NAC-resistant (Figure 2B), suggesting that about 80% of the genes deleted in the isolated strains may be involved in ROS homeostasis. 

**Figure 4:**
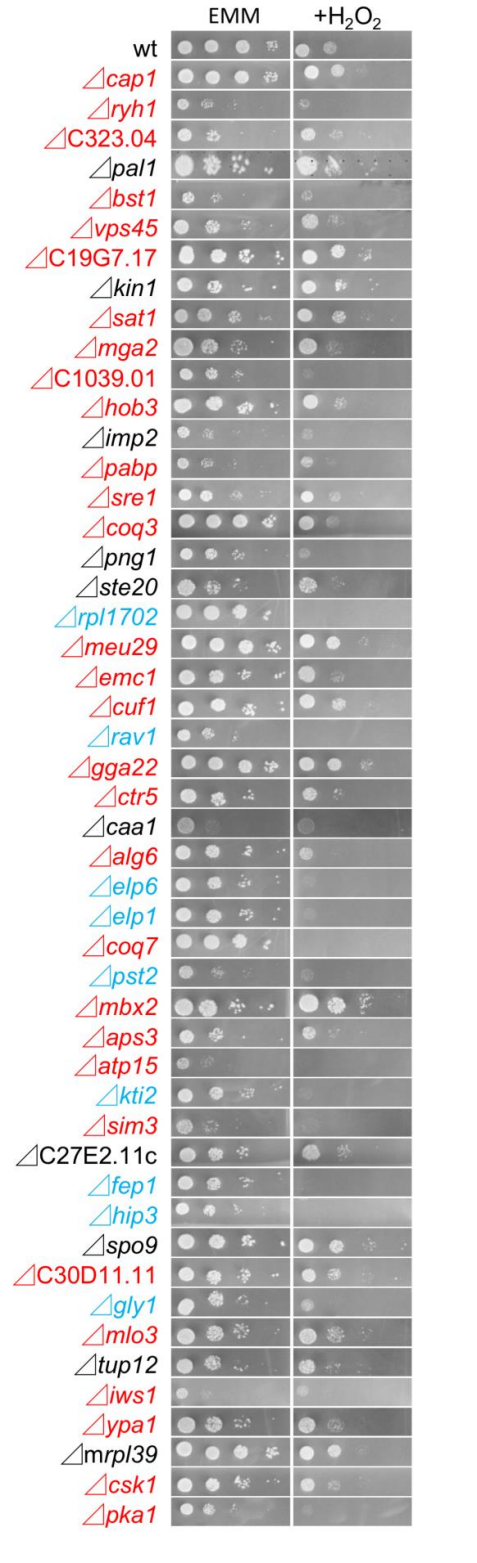
FIGURE 4: H_2_O_2_-sensitivity screening using dilution-series spot assay for the 49 KO strains identified in the third screen. Raw data of chemical genomics screening on the EMM plate with 2 mM H_2_O_2_ (EMM_, _+H_2_O_2_).

We also investigated the growth of the 49 FTY720-sensitive mutants in an Edinburgh minimal medium (EMM) plate containing 2 mM H_2_O_2_, to examine the existence of the cross-hypersensitivity between FTY720 and H_2_O_2_ (Figure 4). The results showed that 9 mutant cells Figure 2B; Group2) also exhibited enhanced sensitivities to H_2_O_2_ as compared with the wt cells (Figure 4; EMM, +H_2_O_2_), whereas 30 mutants (Figure 2B; Group1) exhibited preferential growth defects induced by FTY720. Interestingly, none of the NAC-irremedial FTY720 sensitive mutants (Figure 2B; Group3) exhibited H_2_O_2_ hypersensitivities.

### FTY720-sensitive gene-deletion cells exhibited deregulated ROS levels in response to FTY720 or H_2_O_2_

**Figure 5:**
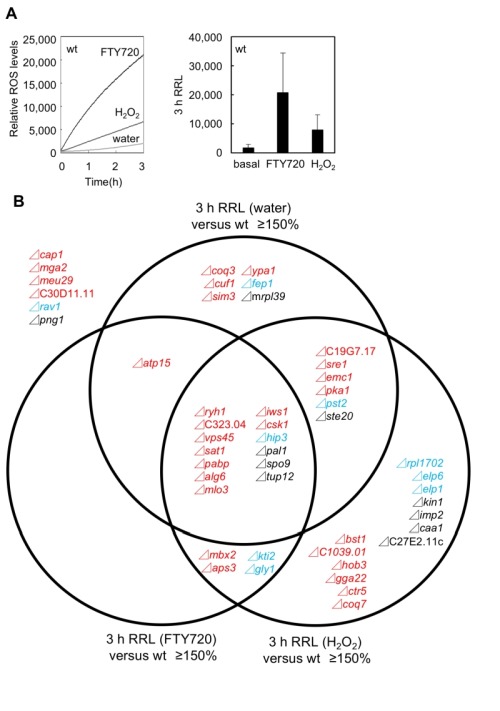
FIGURE 5: Summary of the difference of ROS production in these FTY720-sensitive mutants. ** (A)** The addition of FTY720 or H_2_O_2_ produced ROS in the wt strain (KP178). Left panel: measurement of ROS production treated with water, 30 μM FTY720 or 2 mM H_2_O_2_. ROS accumulation was measured for 3 h. The data shown are representative of multiple experiments. Right panel: Histograms show the average of peak heights from three independent experiments shown in the left panel. Error bars, SD. **(B)** Venn diagram showing the overlap between mutants that exhibited higher ROS levels (defined as >150% of RRL (Relative ROS Levels) of the wt strain) in the absence or presence of FTY720 or H_2_O_2_ treatment for 3 hr (Figure 6).

The above results prompted us to investigate the actual involvement of ROS in mediating the FTY720 hyper-sensitivity in these mutant cells. For this purpose, the levels of ROS in the 49 FTY720-sensitive mutants in comparison with that of the wt cells in response to FTY720 or H_2_O_2_ were analyzed using the general ROS probe DCF-DA. Consistent with our previous data using adenine plus wt cells (HM123: *h^- ^leu1-32*) 23, both FTY720 and H_2_O_2_ treatment stimulated ROS production in the adenine-requiring wt cells (KP178; *h^-^ leu1-32 ura4-D18 ade6-M210 *Figure 5A). It should be noted that 30 µM FTY720 markedly stimulated ROS production, which was more prominent than that achieved by 2 mM H_2_O_2_, consistent with the notion that FTY720 is a potent inducer of ROS.

**Figure 6:**
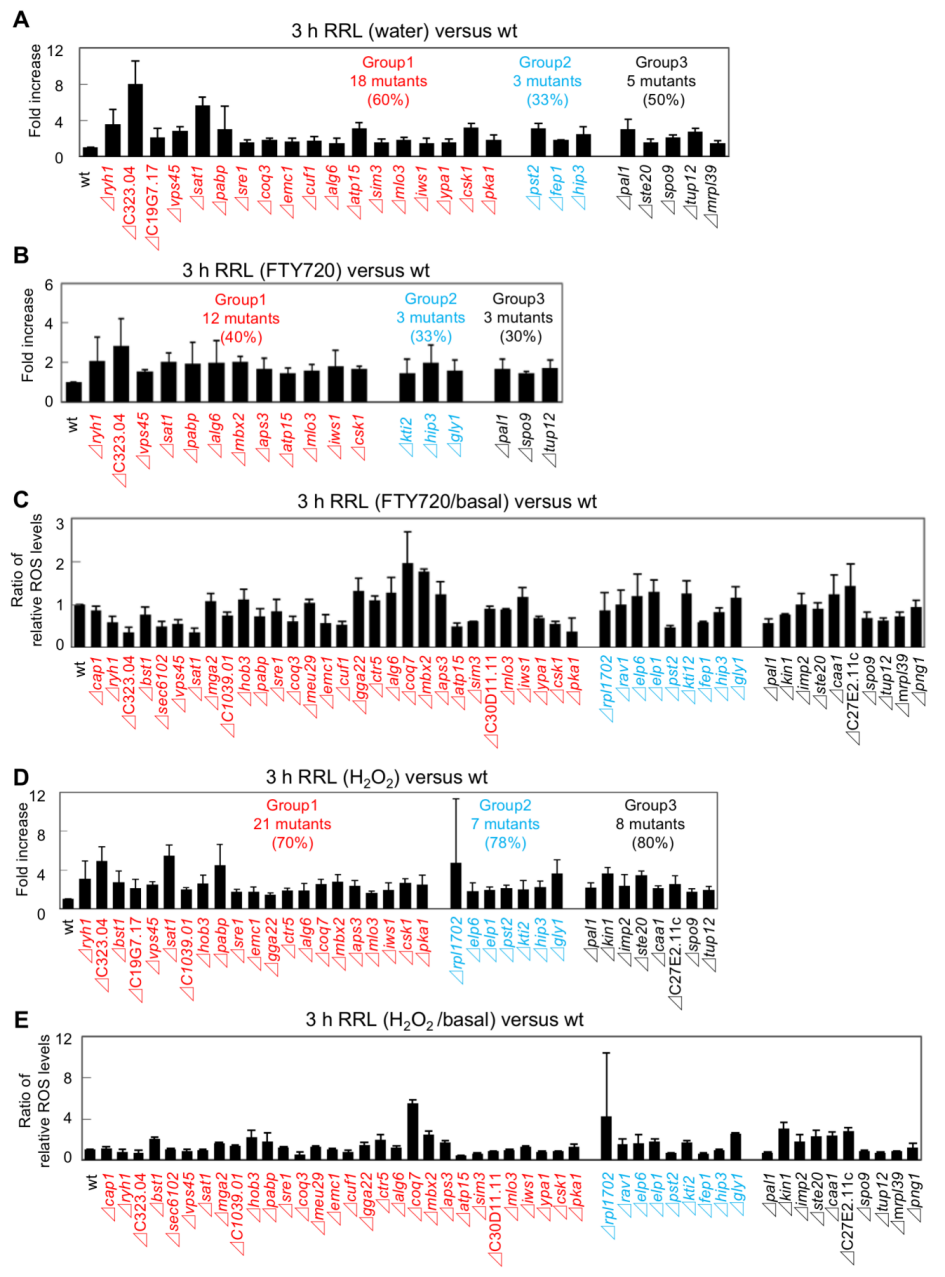
FIGURE 6: FTY720-sensitive gene-deletion cells exhibited deregulated ROS levels in the absence/presence of FTY720 or H_2_O_2_. **(A)** The RRL in the 26 FTY720-sensitive mutants displayed 150% higher than that in the wt strain in the absence of any ROS-inducing agents. **(B)** The RRL in the 18 FTY720-sensitive mutants displayed 150% higher than that in the wt strain upon FTY720 treatment. **(C)** Ratios of ROS levels between after and before the FTY720 stimuli in comparison with those of the wt strain. **(D)** The RRL in the 36 FTY720-sensitive mutants displayed 150% higher than that in the wt strain upon H_2_O_2_ treatment. **(E)** Ratios of ROS levels between after and before the H_2_O_2_ stimuli in comparison with those of the wt strain. These ROS accumulations were measured for 3 h. The data were averaged from three independent experiments. Error bars, SD.

We first focused on the basal levels (water) of ROS production in the FTY720-sensitive mutants in comparison with that of the wt cells. Out of the 49 FTY720-sensitive strains, 26 (53%) exhibited significantly higher ROS levels (defined as ≥150% of that of the wt cells) in the absence of any ROS-inducing agents (Figure 5B: pattern1). Notably, among the 30 mutants, 18 (60%) in Group 1 were included in this pattern, whereas 3 mutants in Group 2 and 5 mutants in Group 3 were included (Figure 6A, S1, water).

We next analyzed the ROS levels after the addition of FTY720 or H_2_O_2_ for 3 hr. Eighteen strains showed significantly higher ROS levels (defined as ≥150% of that of the wt strain) upon FTY720 treatment for 3 hr (Figure 5B). Twelve mutants in Group 1 were included in this pattern (Figure 6B, S2), and 10 mutants (Δ*ryh1*, Δ*SPAC323.04*, Δ*vps45*, Δ*sat1*, Δ*pabp*, Δ*alg6*, Δ*atp15*, Δ*mlo3*, Δ*iws1*, and Δ*csk1*) except for Δ*mbx2* and Δ*aps3 *in Group 1 overlapped with the strains included in the strain list in Pattern 1 (Figure 5B). Thus, these 10 genes are likely to be involved in maintaining ROS homeostasis at steady-state levels and their FTY720 sensitivities may be explained due to excess amounts of ROS accumulation upon FTY720 treatment.

Furthermore, the ROS levels after the addition of H_2_O_2_ for 3 hr were analyzed. Notably, among the 49 FTY720 sensitive mutants, 36 (73%) exhibited higher ROS production upon H_2_O_2_ treatment, and 21 (70%) out of 30 mutants in Group 1 were included in this pattern (Figure 6D, S3). Notably, 7 out of 9 mutants (78%) in Group 2 were included in this pattern, and 3 of them (Δ*kti2*, Δ*hip3*, Δ*gly1*) also exhibited higher ROS levels upon FTY720 treatment (Pattern 2).

Finally, the ratio of ROS levels between before and after the FTY720 or H_2_O_2_ treatment in comparison with those of the wt strains was analysed (Figure 6C, 6E). Both Δ*coq7 *and Δ*mbx2 *strains exhibited the highest ratios upon FTY720 and H_2_O_2_ treatment (2.0 and 1.8 upon FTY720 and 5.5 and 2.4 upon H_2_O_2_, respectively). Thus, Coq7 and Mbx2 may be required for the adaptation mechanisms to combat acute damages in response to ROS-inducing stimuli.

## DISCUSSION

Our present genome-wide study revealed genes important for determining sensitivity to FTY720. In budding yeast, it has been reported that ubiquitin pathway proteins are involved in the mechanism of action of FTY720 25. In fission yeast, several genes required for proper calcium homeostasis, as well as genes required for stress responses are required for tolerance to FTY720 22. However, no reports have been made on a global view of the cellular mechanisms required to cope with the toxicity and to determine the sensitivity of FTY720.

Several lines of evidence from the study suggested the involvement of ROS in the mechanisms of FTY720-induced growth defects in the 49 isolated mutants. First, the growth of the 39 mutants (Group 1 and Group 2) can be alleviated by the addition of the antioxidant NAC. Second, 9 mutants (Group 2) exhibited cross-sensitivity to H_2_O_2_. Third, out of the 49 FTY720-sensitive mutants, 26 (53%) displayed elevated steady-state levels of ROS. Finally, 37 mutants displayed significant increase in ROS levels in response to FTY720 or H_2_O_2_ in comparison to the wt strains. These data point towards oxidative stress as a major mechanism of the FTY720 sensitivities observed.

In the *S. pombe* genome database (http://pombase-preview.babraham.ac.uk , the coding genes were categorized using the Gene Ontology (GO) slim terms (https://www.pombase.org/browse-curation/fission-yeast-go-slim-terms). We then mapped the intracellular localization of the 49 gene products identified in our screen (Figure 3), according to the GO slim terms and ORFeome information 26. Notably, impressive numbers of cellular processes associated with the identified FTY720-sensitive gene products were functionally enriched in the nucleus, wherein "Gene expression" related processes ranging from transcription [GO:0006351; *mga2^+^, sre1^+^, cuf1^+^, mbx2^+^, iws1^+^, csk1^+^, pka1^+^, fep1^+^, tup12^+^*], chromatin organization [GO:0006325; *sim3^+^*, *hip3^+^*,* pst2^+^*, *png1^+^*], tRNA metabolic process [GO:0006399; *elp6^+^*, *elp1^+^*, *kti2^+^*], to the nucleocytoplasmic transport [GO:0006913; *pabp^+^*, *mlo3^+^*]) were executed.

Why are these mutant cells defective in gene expression processes involved in ROS homeostasis? Interestingly, the *elp1^+ ^*and *elp6^+^* genes, which are involved in tRNA metabolic processes, encode elongator complex subunits, and *kti2^+^* encodes a regulator of the elongator complex which interacts with subunits of the elongator complex 27. Elongator is an evolutionary highly conserved complex and has been reported to be a histone acetyltransferase complex involved in the elongation of RNA polymerase II transcription. In budding yeast, mutations in any of the six Elongator subunit genes (*ELP1-6*) or its regulators (*KTI11-13*) induce similar phenotypes, and the *ELP1-6* and *KTI11-13* genes are all required for an early step in synthesis of 5-methoxycarbonylmethyl (mcm^5^) and 5-carbamoylmethyl (ncm^5^) groups present on uridines at the wobble position in tRNAs 27. Jorge Fernádez-Vázquez *et al., *reported that Sin3/Elp3, an Elongator component in fission yeast is required for tolerance to H_2_O_2_ by tRNA modification 28. The lack of a functional elongator complex results in oxidative stress phenotypes due to its contribution to tRNA modification and subsequent translation inefficiency of certain stress-induced, highly expressed mRNAs governed by the stress-activated protein kinase (SAPK) Sty1-Atf1-Pcr1 pathway 28. The Sty1/Atf1 signaling pathway plays a critical role in oxidative stress responses by regulating gene expression of various antioxidative enzymes, such as *ctt1^+^*which encodes a catalase and *sod1^+^*which encodes a superoxide dismutase. Therefore, structural components and regulators of the elongator complex as well as the gene products identified in our screen involved in "gene expression processes" may be functionally related to FTY720 and/or H_2_O_2_-mediated oxidative stress responses, presumably by affecting gene expression governed by the SAPK/Atf1/Pcr1 pathway and that of antioxidative enzymes.

### The role of mitochondria in FTY720 tolerance

Six gene products (Coq3, Coq7, SPAC323.04, Atp15, SPAC27E2.11c, and Mrpl39) were mapped to localize at the mitochondria, which is the main source of ROS in most cell types. Of these, Coq3 and Coq7 are involved in ubiquinone biosynthetic processes and are required for the last step of CoQ10 synthesis, which is essential for electron transport in the mitochondrial respiratory chain and antioxidant defense 29,30,31. Zuin *et al*., reported that mitochondrial dysfunction increases oxidative stress in fission yeast 32. Additionally, they found that mitochondria mutants that are defective in ubiquinone synthesis are more sensitive to intrinsic and extrinsic oxidative stress than other mitochondrial mutants, as ubiquinone deficiency enhances ROS production and impairs cellular antioxidant capacity 32. Atp15 and SPAC323.04 are a mitochondrial proton-transporting ATP synthase and a mitochondrial ATPase, respectively. Additionally, they found that mitochondrial mutants require antioxidants for growth on defined medium and suggested that they are more prone to produce ROS than a wt strain, consistent with our data that Coq3, SPAC323.04 and Atp15 belong to Group 1 which displayed increased steady-state ROS levels.

### Golgi/Endosomes as determinants of the FTY720-mediated oxidative responses 

It should be noted that several gene products are mapped to localize at the Golgi/Endosome structures (Figure 3). Although the Golgi complex is an organelle that processes proteins and lipids, recent papers have highlighted the importance of the Golgi apparatus as an oxidative stress-associated subcellular organelle. This view is based on several lines of evidence showing that 1) oxidative stress induces Golgi fragmentation 33, 2) ubiquinone (or coenzyme Q) is enriched in the Golgi 34, and that 3) the Golgi is a key organelle in maintaining Ca^2+^ homeostasis, which critically influences oxidative stress responses and apoptosis 35,36. Intriguingly, our previous data demonstrated that FTY720 stimulates both ROS accumulation and an intracellular increase in Ca^2+^ concentrations, and that the addition of NAC significantly neutralized ROS accumulation as well as intracellular Ca^2+^ increase upon FTY720 treatment 22,23. Thus, the identified gene products which localize at the Golgi/Endosome structures may affect the cytoplasmic Ca^2+^ concentrations via their involvement in the membrane trafficking processes (Ryh1, Gga22, and Vps45) or by direct regulation of Ca^2+^ homeostasis (Meu29 Ca^2+^ transporter). Deterioration of these processes, in combination with ROS accumulation, may explain the FTY720 sensitivities associated with these gene deletion strains.

### Lipid homeostasis and FTY720 sensitivities

Finally, another category of cellular function associated with FTY720-sensitive genes is "lipid metabolic process" (Mga2, Bst1, Alg6, Spo9 and Sre1) (Table S1, Figure 3). Of these, Mga2 and Sre1 are transcription factors, functionally homologous to mammalian SREBP-1 and SREBP-2, respectively, both of which regulate the lipid homeostasis pathway in an oxygen-responsive manner 37,38. Interestingly, in the budding yeast Mga2 is essential for an adaptive response to oxidative stress (H_2_O_2_ treatment) and Sre1 activation is induced by H_2_O_2_, thus indicating a strong functional connection between these transcription factors and lipid homeostasis in response to oxidative stress 37,38,39. Because lipids are important in the post-translational modification of ROS and the peroxidation of membrane lipids leads to the loss of membrane fluidity and elasticity, impaired cellular function, excess ROS accumulation and lipid dyshomeostasis in these deletion strains may explain their hypersensitivity to FTY720 treatment.

### What is the physiological significance of FTY720-induced ROS in a therapeutic context of cancer treatment?

Oxidative stress has generated significant interest because it is a major contributor to the etiology of severe pathologies, including inflammation and cancer 40. In several cancer cell types, FTY720 increases ROS generation, which then results in cell death. In mantle cell lymphoma, ROS is upstream of cell death induced by FTY720 15. In multiple myeloma cells, FTY720-induced ROS activity promotes autophagy, reduces the expression of Mcl-1, Survivin, Bcl-2 and increases cleavage of Bid, ultimately leading to apoptosis of multiple myeloma cells 16. Although it remains controversial if FTY720 exerts antitumor activity independently of its S1P modulating activity, in many cases phosphorylation of FTY720 is not required for its anti-cancer property, indicating the involvement of S1PR-independent mechanisms which seem to be different from the immunosuppressive property of FTY720 41. In addition, FTY720 and OSU-2S, a non-immunosuppressive analogue of FTY720, regulates antitumor effects* via activating NADPH oxidase by up-regulating the gp91^phox^ subunit expression, and subsequently activating *PKCδ-caspase-3 signaling in hepatocellular carcinoma 11. Consistently, we also compared ROS stimulating activity between FTY720 and the phosphorylated form of FTY720 (FTY720-P). Our results show that FTY720-P failed to stimulate ROS production (Figure S4). Given the findings that the fission yeast genome does not express the S1P receptor and that this organism is an excellent model to analyze stress responses and ROS-mediating signaling pathways, fission yeast may contribute to revealing novel determinants as well as the mode of action for FTY720 activity as an anti-cancer compound.

Recently, several oxidation therapies for cancer treatment are attracting attention. Cancer stem cells possess mechanisms to protect themselves from ROS, thereby making them resistant to chemotherapies 42,43. Recent studies on chemical compounds such as xCT inhibitor or CD44 antibodies were successful in targeting this defense mechanisms which stimulate ROS in a cancer-cell specific manner 44,45. Therefore, FTY720 might serve as an excellent cancer therapy, when combined with targeting ROS defense mechanisms. In animal models, the doses required for the anticancer effects of FTY720 (5 or 10 mg/kg/day) are higher than those used in multiple sclerosis models (< 0.5 mg/kg/day) 8,9,46,47. In this regard, the list of the FTY720 sensitive genes obtained from our screen points to new potential targets for combination therapy as a means to enhance the antitumor efficacy of FTY720.

On the other hand, the information on the FTY720-sensitive genes derived from this study could be connected to the side-effects of Fingolimod. Fingolimod can cause side effects and in rare cases, they can be serious. Side effects from the first dose include low blood pressure and bradycardia. The more common side effects that can occur after the second and other follow-up doses can include infectious diseases, macular edema, diarrhea, coughing, headaches, hair loss, depression, muscle weakness, and dry and itchy skin.

Recently, Gorlino *et al.,* reported that S1P participates in neutrophil trafficking from tissues through lymph and also from blood into lymph nodes by direct action on S1PRs expressed on neutrophils 48. Intriguingly, a congenital neutrophil defect syndrome has been reported to be associated with mutations in VPS45 49, a homologue of the fission yeast Vps45 identified by our study as an FTY720-sensitive gene. In this regard, special caution should be taken when patients harboring mutations in VPS45 are treated with Fingolimod. Thus, the list of causative genes in our study contributes to the emerging topic of personalized medicine and provides valuable information towards the fingolimod-treated patients.

## MATERIALS AND METHODS

### Chemicals

FTY720, Hydrogen peroxide (H_2_O_2_) and N-acetylcysteine (NAC) were purchased from Cayman Chemical Company (Michigan, USA), Wako Pure Chemical Industries, Ltd. (Osaka, Japan) and nacalai tesque (Kyoto, Japan), respectively. Yeast growth media containing each of these chemicals were prepared by mixing stock solutions of these chemicals with the YES or EMM medium to achieve the desired drug concentration. For agar media, the stock solution of the appropriate drug was added after autoclaving and cooling of the media to approximately 50°C.

### Deletion library construction, media methods

Fission yeast haploid gene deletion library [Bioneer version 4.0 (more information is provided at the Bioneer web page, https://us.bioneer.com/products/spombe/spombeliterature.aspx)] 24 was used for genome-wide screens with the supplied wt (KP178; *h^-^ leu1-32 ura4-D18 ade6-M210*) as negative controls. YES (rich yeast extract with supplements) plates are supplemented with 250 mg/L adenine, histidine, leucine, uracil and lysine. In Supplementary Figure 4, HM123 cells (*h^-^ leu1-32*) were used.

### Deletion library screens for FTY720 sensitivity

The deletion library was provided on agar plates and stamped in a 96-well format. We compared the growth of the wt cells using a library of 3,400 deletion mutant cells on YES plus FTY720 plates. The logarithmic phase cells were spotted in quadruplicates (by a Singer ROTOR© robot) onto YES plates containing 0, 10 or 20 μM FTY720 and incubated at 27°C for 4 days for preliminary screen. Deletion mutants that exhibited growth inhibition with 10 or 20 μM FTY720 (1,335 deletion strains) were selected to carry out the secondary screen using a representative streak assay of logarithmic phase cultures of each strain onto YES plates containing 0, 10 or 20 μM FTY720. We carried out these retests in duplicates. The severity of growth inhibition by FTY720 was scored as follows: severe (-3) indicating that the cells completely failed to grow on the plate containing 10 μM FTY720, moderate (-2) indicating that tiny colonies were observed to grow on the same concentrations of the FTY720-containing plate, and mild (-1) indicating that colonies were observed to grow on the 20 μM FTY720-containing plates; however, the size of the colonies was significantly smaller than that of the wt cells. The secondary screen evaluated 134 mutants as sensitive. The rest of the mutants did not show clear growth defects with even 20 μM FTY720 in the single-colony streak assay. The tertiary screen was performed to determine the degree of the sensitivity associated with 134 of the FTY720-sensitive mutants, using a dilution-series spot test. The wt cells and selected mutants were grown to saturation in liquid medium YES at 27°C. The cultures were then resuspended in fresh YES medium to give an optical density (OD) at 660 nm of 0.4 and serially diluted to concentrations of 1×10^-1^ to 1×10^-4^. The 5 μL samples of 10-fold serial dilutions of each yeast cell culture were spotted onto YES plates containing 0, 10 or 20 μM FTY720 and incubated at 27°C for 4 days. The severity of the growth inhibition by FTY720 was scored 3 grades according to comparison (1) between the number of spots that grew on the FTY720-containing YES plates with the number of spots that grew on the FTY720-free YES plates and (2) between the wt and the mutant strains.

### Detection of ROS

Measurement of ROS production in *S. pombe* cells was determined using a previously described method 23. After preincubation of the yeast cells (OD_660_ nm = 0.4) in EMM medium with 40 μM DCF-DA (Sigma-Aldrich, Missouri, St. Louis, MO, USA) at 30°C for 60 min, the cell suspensions were withdrawn and then washed with phosphate-buffered saline (PBS) twice, resuspended in PBS, and treated with water, 30 μM FTY720 or 2 mM H_2_O_2_, as indicated. Fluorescence intensity was analyzed by fluorescence spectrophotometer (Infinite 200 PRO series; Tecan, Switzerland) with excitation at 480 nm and emission at 530 nm at 1-min intervals.

### Statistical analysis

The mean and the standard deviation (SD) of the RLU values for three different experiments were calculated for each sample. Data were analyzed using a one-way ANOVA, followed by a post hoc test using Williams’ test (Figure 1B).

## SUPPLEMENTAL MATERIAL

Click here for supplemental data file.

All supplemental data for this article are also available online at  http://microbialcell.com/researcharticles/a-genome-wide-screen-for-tfy720-sensitive-mutants-reveals-genes-required-for-ros-homeostasis/.

## References

[1] Adachi K, Kohara T, Nakao N, Arita M, Chiba K, Mishina T, Sasaki S, Fujita T (1995). Design, synthesis, and structure-activity relationships of 2-substituted-2-amino-1,3-propanediols:discovery of a novel immunosuppressant, FTY720.. Bio Med Chem Lett.

[2] Strader CR, Pearce CJ, Oberlies NH (2011). Fingolimod (FTY720): a recently approved multiple sclerosis drug based on a fungal secondary metabolite.. J Nat Prod.

[3] Azuma H, Takahara S, Ichimaru N, Wang JD, Itoh Y, Otsuki Y, Morimoto J, Fukui R, Hoshiga M, Ishihara T, Nonomura N, Suzuki S, Okuyama A, Katsuoka Y (2002). Marked prevention of tumor growth and metastasis by a novel immunosuppressive agent, FTY720, in mouse breast cancer models.. Cancer Res.

[4] Nagaoka Y, Otsuki K, Fujita T, Uesato S (2008). Effects of phosphorylation of immunomodulatory agent FTY720 (fingolimod) on antiproliferative activity against breast and colon cancer cells.. Biol Pharm Bull.

[5] Hait NC, Avni D, Yamada A, Nagahashi M, Aoyagi T, Aoki H, Dumur CI, Zelenko Z, Gallagher EJ, Leroith D, Milstien S, Takabe K, Spiegel S (2015). The phosphorylated prodrug FTY720 is a histone deacetylase inhibitor that reactivates ERα expression and enhances hormonal therapy for breast cancer.. Oncogenesis.

[6] Estrada-Bernal A1, Palanichamy K, Ray Chaudhury A, Van Brocklyn JR (2012). Induction of brain tumor stem cell apoptosis by FTY720: a potential therapeutic agent for glioblastoma.. Neuro Oncol.

[7] Zhang L, Wang H, Zhu J, Ding K, Xu J (2014). FTY720 reduces migration and invasion of human glioblastoma cell lines via inhibiting the PI3K/AKT/mTOR/p70S6K signaling pathway.. Tumour Biol.

[8] Zhang L, Wang H, Ding K, Xu J (2015). FTY720 induces autophagy-related apoptosis and necroptosis in human glioblastoma cells.. Toxicol Lett.

[9] Lee TK, Man K, Ho JW, Wang XH, Poon RT, Xu Y, Ng KT, Chu AC, Sun CK, Ng IO, Sun HC, Tang ZY, Xu R, Fan ST (2005). FTY720: a promising agent for treatment of metastatic hepatocellular carcinoma.. Clin Cancer Res.

[10] Hung JH, Lu YS, Wang YC, Ma YH, Wang DS, Kulp SK, Muthusamy N, Byrd JC, Cheng AL, Chen CS (2008). FTY720 induces apoptosis in hepatocellular carcinoma cells through activation of protein kinase C delta signaling.. Cancer Res.

[11] Omar HA, Chou CC, Berman-Booty LD, Ma Y, Hung JH, Wang D, Kogure T, Patel T, Terracciano L, Muthusamy N, Byrd JC, Kulp SK, Chen CS (2011). Antitumor effects of OSU-2S, a nonimmunosuppressive analogue of FTY720, in hepatocellular carcinoma.. Hepatology.

[12] Szymiczek A, Pastorino S, Larson D, Tanji M, Pellegrini L, Xue J, Li S, Giorgi C, Pinton P, Takinishi Y, Pass HI, Furuya H, Gaudino G, Napolitano A, Carbone M, Yang H (2017). FTY720 inhibits mesothelioma growth in vitro and in a syngeneic mouse model.. J Transl Med.

[13] Zhang N, Qi Y, Wadham C, Wang L, Warren A, Di W, Xia P (2010). FTY720 induces necrotic cell death and autophagy in ovarian cancer cells: a protective role of autophagy..

[14] Wallington-Beddoe CT, Hewson J, Bradstock KF, Bendall LJ (2011). FTY720 produces caspase-independent cell death of acute lymphoblastic leukemia cells.. Autophagy.

[15] Liu Q, Alinari L, Chen CS, Yan F, Dalton JT, Lapalombella R, Zhang X, Mani R, Lin T, Byrd JC, Baiocchi RA, Muthusamy N (2010). FTY720 shows promising in vitro and in vivo preclinical activity by downmodulating Cyclin D1 and phospho-Akt in mantle cell lymphoma.. Clin Cancer Res.

[16] Liao A, Hu R, Zhao Q, Li J, Li Y, Yao K, Zhang R, Wang H, Yang W, Liu Z (2012). Autophagy induced by FTY720 promotes apoptosis in U266 cells.. Eur J Pharm Sci.

[17] Miyatake M, Kuno T, Kita A, Katsura K, Takegawa K, Uno S, Nabata T, Sugiura R (2007). Valproic acid affects membrane trafficking and cell-wall integrity in fission yeast.. Genetics.

[18] Sugiura R, Kita A, Tsutsui N, Muraoka O, Hagihara K, Umeda N, Kunoh T, Takada H, Hirose D (2012). Acremomannolipin A, the potential calcium signal modulator with a characteristic glycolipid structure from the filamentous fungus Acremonium strictum.. Bioorg Med Chem Lett.

[19] Kita A, Higa M, Doi A, Satoh R, Sugiura R (2015). Imp2, the PSTPIP homolog in fission yeast, affects sensitivity to the immunosuppressant FK506 and membrane trafficking in fission yeast.. Biochem Biophys Res Commun.

[20] Doi A, Fujimoto A, Sato S, Uno T, Kanda Y, Asami K, Tanaka Y, Kita A, Satoh R, Sugiura R (2015). Chemical genomics approach to identify genes associated with sensitivity to rapamycin in the fission yeast Schizosaccharomyces pombe.. Genes Cells.

[21] Satoh R, Hagihara K, Matsuura K, Manse Y, Kita A, Kunoh T, Masuko T, Moriyama M, Moriyama H, Tanabe G, Muraoka O, Sugiura R (2017). Identification of ACA-28, a 1'-acetoxychavicol acetate analogue compound, as a novel modulator of ERK MAPK signaling, which preferentially kills human melanoma cells.. Genes Cell.

[22] Hagihara K, Kita A, Mizukura A, Yao M, Kitai Y, Kunoh T, Masuko T, Matzno S, Chiba K, Sugiura R (2013). Fingolimod (FTY720) stimulates Ca(2+)/calcineurin signaling in fission yeast.. PLoS One.

[23] Hagihara K, Mizukura A, Kitai Y, Yao M, Ishida K, Kita A, Kunoh T, Masuko T, Matzno S, Chiba K, Sugiura R (2014). FTY720 stimulated ROS generation and the Sty1/Atf1 signaling pathway in the fission yeast Schizosaccharomyces pombe.. Genes Cells.

[24] Kim DU, Hayles J, Kim D, Wood V, Park HO, Won M, Yoo HS, Duhig T, Nam M, Palmer G, Han S, Jeffery L, Baek ST, Lee H, Shim YS, Lee M, Kim L, Heo KS, Noh EJ, Lee AR, Jang YJ, Chung KS, Choi SJ, Park JY, Park Y, Kim HM, Park SK, Park HJ, Kang EJ, Kim HB (2010). Analysis of a genome-wide set of gene deletions in the fission yeast Schizosaccharomyces pombe.. Nat Biotechnol.

[25] Welsch CA, Hagiwara S, Goetschy JF, Movva NR (2003). Ubiquitin pathway proteins influence the mechanism of action of the novel immunosuppressive drug FTY720 in Saccharomyces cerevisiae.. J Biol Chem.

[26] Matsuyama A, Arai R, Yashiroda Y, Shirai A, Kamata A, Sekido S, Kobayashi Y, Hashimoto A, Hamamoto M, Hiraoka Y, Horinouchi S, Yoshida M (2006). ORFeome cloning and global analysis of protein localization in the fission yeast Schizosaccharomyces pombe.. Nat Biotechnol.

[27] Huang B, Johansson MJ, Bystrom AS (2005). An early step in wobble uridine tRNA modification requires the Elongator complex.. RNA.

[28] Fernández-Vázquez J, Vargas-Pérez I, Sansó M, Buhne K, Carmona M, Paulo E, Hermand D, Rodríguez-Gabriel M, Ayté J, Leidel S, Hidalgo E (2013). Modification of tRNA(Lys) UUU by elongator is essential for efficient translation of stress mRNAs.. PLoS Genet.

[29] Hayashi K, Ogiyama Y, Yokomi K, Nakagawa T, Kaino T, Kawamukai M (2014). Functional conservation of coenzyme Q biosynthetic genes among yeasts, plants, and humans.. PLoS One.

[30] Miki R, Saiki R, Ozoe Y, Kawamukai M (2008). Comparison of a coq7 deletion mutant with other respiration-defective mutants in fission yeast.. FEBS J.

[31] Quinzii CM, López LC, Gilkerson RW, Dorado B, Coku J, Naini AB, Lagier-Tourenne C, Schuelke M, Salviati L, Carrozzo R, Santorelli F, Rahman S, Tazir M, Koenig M, DiMauro S, Hirano M (2010). Reactive oxygen species, oxidative stress, and cell death correlate with level of CoQ10 deficiency.. FASEB J.

[32] Zuin A, Gabrielli N, Calvo IA, García-Santamarina S, Hoe KL, Kim DU, Park HO, Hayles J, Ayté J, Hidalgo E (2008). Mitochondrial dysfunction increases oxidative stress and decreases chronological life span in fission yeast.. PLoS One.

[33] Jiang Z1, Hu Z, Zeng L, Lu W, Zhang H, Li T, Xiao H (2011). The role of the Golgi apparatus in oxidative stress: is this organelle less significant than mitochondria?. Free Radic Biol Med.

[34] Crane FL (2001). Biochemical functions of coenzyme Q10.. J Am Coll Nutr.

[35] Fabrizio de Mattia, Caroline Gubser, Michiel MT van Dommelen, Henk-Jan Visch, Felix Distelmaier, Antonio Postigo, Tomas Luyten, Jan B Parys, Humbert de Smedt, Geoffey L Smith, Peter HGM Willems, Frank JM van Kuppeveld (2009). Human Golgi Antiapoptotic Protein Modulates Intracellular Calcium Fluxes.. Mol Biol Cell.

[36] Miseta A, Fu L, Kellermayer R, Buckley J, Bedwell DM (1999). The Golgi apparatus plays a significant role in the maintenance of Ca2+ homeostasis in the vps33Delta vacuolar biogenesis mutant of Saccharomyces cerevisiae.. J Biol Chem.

[37] Hughes AL, Lee CY, Bien CM, Espenshade PJ (2007). 4-Methyl sterols regulate fission yeast SREBP-Scap under low oxygen and cell stress.. J Biol Chem.

[38] Burr R, Stewart EV, Shao W, Zhao S, Hannibal-Bach HK, Ejsing CS, Espenshade PJ (2016). Mga2 Transcription Factor Regulates an Oxygen-responsive Lipid Homeostasis Pathway in Fission Yeast.. J Biol Chem.

[39] Kelley R, Ideker T (2009). Genome-wide fitness and expression profiling implicate Mga2 in adaptation to hydrogen peroxide..

[40] Reuter S, Gupta SC, Chaturvedi MM, Aggarwal BB (2010). Oxidative stress, inflammation, and cancer: how are they linked?. Free Radic Biol Med.

[41] Vadas M, Xia P, McCaughan G, Gamble J (2008). The role of sphingosine kinase 1 in cancer: oncogene or non-oncogene addiction?. Biochim Biophys Acta.

[42] Phillips TM1, McBride WH, Pajonk F (2006). The response of CD24(-/low)/CD44+ breast cancer-initiating cells to radiation.. J Natl Cancer Inst.

[43] Suzuki S, Okada M, Shibuya K, Seino M, Sato A, Takeda H, Seino S, Yoshioka T, Kitanaka C (2015). JNK suppression of chemotherapeutic agents-induced ROS confers chemoresistance on pancreatic cancer stem cells.. Oncotarget.

[44] Ishimoto T, Nagano O, Yae T, Tamada M, Motohara T, Oshima H, Oshima M, Ikeda T, Asaba R, Yagi H, Masuko T, Shimizu T, Ishikawa T, Kai K, Takahashi E, Imamura Y, Baba Y, Ohmura M, Suematsu M, Baba H, Saya H (2011). CD44 variant regulates redox status in cancer cells by stabilizing the xCT subunit of system xc(-) and thereby promotes tumor growth.. Cancer Cell.

[45] Yae T, Tsuchihashi K, Ishimoto T, Motohara T, Yoshikawa M, Yoshida GJ, Wada T, Masuko T, Mogushi K, Tanaka H, Osawa T, Kanki Y, Minami T, Aburatani H, Ohmura M, Kubo A, Suematsu M, Takahashi K, Saya H, Nagano O (2012). Alternative splicing of CD44 mRNA by ESRP1 enhances lung colonization of metastatic cancer cell.. Nat Commun.

[46] Kataoka H, Sugahara K, Shimano K, Teshima K, Koyama M, Fukunari A, Chiba K (2005). FTY720, sphingosine 1-phosphate receptor modulator, ameliorates experimental autoimmune encephalomyelitis by inhibition of T cell infiltration.. Cell Mol Immunol.

[47] Zhang L, Wang HD, Ji XJ, Cong ZX, Zhu JH, Zhou Y (2013). FTY720 for cancer therapy (Review).. Oncol Rep.

[48] Gorlino CV, Ranocchia RP, Harman MF, García IA, Crespo MI, Morón G, Maletto BA, Pistoresi-Palencia MC (2014). Neutrophils exhibit differential requirements for homing molecules in their lymphatic and blood trafficking into draining lymph nodes.. J Immunol.

[49] Vilboux T, Lev A, Malicdan MC, Simon AJ, Järvinen P, Racek T, Puchalka J, Sood R, Carrington B, Bishop K, Mullikin J, Huizing M, Garty BZ, Eyal E, Wolach B, Gavrieli R, Toren A, Soudack M, Atawneh OM, Babushkin T, Schiby G, Cullinane A, Avivi C, Polak-Charcon S, Barshack I, Amariglio N, Rechavi G, van der Werff ten Bosch J, Anikster Y, Klein C (2013). A congenital neutrophil defect syndrome associated with mutations in VPS45.. N Engl J Med.

