# Supplementary material for: A genome-wide screen for FTY720-sensitive mutants reveals genes required for ROS homeostasis
Source: Microb Cell. 2017 Nov 27;4(12):390–401. doi: 10.15698/mic2017.12.601 (PMC5722642; doi:10.15698/mic2017.12.601)
Supplement: Supplementary file 1 [file mic-04-390-s01.pdf]

Table S1. List of *Schizosaccharomyces pombe* FTY720-sensitive mutants identified in the chemical genomics screen.

S. pombe	10 μM	20 μM	*S. cerevisiae*	*H. sapiens*	Product	Representative GO slim
mga2	-2.5	-4	MGA2, SPT23	-	IPT/TIG ankyrin repeat containing transcription regulator of fatty acid biosynthesis	lipid metabolic process
sre1	-2	-3	-	SREBF1	sterol regulatory element binding protein, transcription factor	lipid metabolic process
alg6	-1.5	-2.5	ALG6	ALG6	glucosyltransferase	lipid metabolic process
spo9	0	-2.5	ERG20	FDPS	farnesyl pyrophosphate synthetase	lipid metabolic process
elp6	-1.5	-2	ELP6	ELP6	elongator complex subunit	tRNA metabolic process
elp1	-1.5	-1.5	IKI3	IKBKAP	elongator subunit	tRNA metabolic process
kti2	-1	-2	KTI12	KTI12	elongator complex associated protein	tRNA metabolic process
caa1	-1.5	-2.5	AAT2	GOT1/L1	cytoplasmic aspartate aminotransferase	cellular amino acid metabolic process
gly1	0	-1.5	GLY1	-	threonine aldolase	cellular amino acid metabolic process
cap1	-4	-4	SRV2	CAP1/2	adenylyl cyclase-associated protein	nucleobase-containing small molecule metabolic process
atp15	-1	-2	ATP15	ATP5E	F0-ATPase epsilon subunit	carbohydrate derivative metabolic process
coq3	-2	-3	COQ3	COQ3	hexaprenyldihydroxybenzoate methyltransferase	cofactor metabolic process
coq7	-1.5	-1.5	CAT5	COQ7	ubiquinone biosynthesis protein	cofactor metabolic process

Table S1 (Continued)

S. pombe	10 μM	20 μM	*S. cerevisiae*	*H. sapiens*	Product	Representative GO slim
cuf1	-2	-2	HAA1, CUP2, MAC1	-	nutritional copper sensing transcription factor	transcription, DNA-templated
mbx2	-1	-2.5	RLM1, SMP1	-	MADS-box transcription factor	transcription, DNA-templated
fep1	-0.5	-2	-	-	iron-sensing transcription factor	transcription, DNA-templated
tup12	0	-1.5	TUP1	-	transcriptional corepressor	transcription, DNA-templated
pka1	0	-1	TPK1/2/3	PRKX, PRKACA/B/G	cAMP-dependent protein kinase catalytic subunit	transcription, DNA-templated
csk1	0	-1	CTK1	-	cyclin-dependent kinase activating kinase	transcription, DNA-templated
png1	-2	-2.5	YNG1, PHO23, YNG2	ING2/3/4/5	ING family homolog	chromatin organization
pst2	-1.3	-1.7	SIN3	SIN3A/B	Clr6 histone deacetylase complex subunit	chromatin organization
sim3	-1	-1.5	HIF1	NASP	NASP family CENP-A chaperone	chromatin organization
hip3	-0.5	-1.5	HIR3	CABIN1	HIRA interacting protein	chromatin organization
iws1	0	-1.5	SPN1	IWS1	transcription elongation factor complex subunit	chromatin organization
rpl1702	-2	-2.5	RPL17A/B	RPL17	60S ribosomal protein L17	cytoplasmic translation
mrpl39	0	-1	MRPL39	MRPL33	mitochondrial ribosomal protein subunit L39	mitochondrial translation
pabp	-2.3	-2.7	PAB1	PABPC1/3/4	mRNA export shuttling protein	nucleocytoplasmic transport

Table S1 (Continued)

S. pombe	10 µM	20 µM	*S. cerevisiae*	*H. sapiens*	Product	Representative GO slim
mlo3	0	-1.5	YRA1	ALYREF	RNA binding protein	nucleocytoplasmic transport
bst1	-3.5	-3.5	BST1	PGAP1	GPI inositol deacylase	vesicle-mediated transport
vps45	-3	-4	VPS45	VPS45	vacuolar sorting protein	vesicle-mediated transport
sat1	-3	-3	RGP1	RGP1	Golgi membrane exchange factor subunit	vesicle-mediated transport
rav1	-2	-2	RAV1	DMXL1/2	RAVE complex subunit	vesicle-mediated transport
gga22	-2	-2	GGA1/2	TOM1/L1/L2	Golgi localized Arf binding gamma-adaptin ortholog	vesicle-mediated transport
aps3	-1	-2	APS3	AP3S1/2	AP-3 adaptor complex subunit	vesicle-mediated transport
C19G7.17	-3	-3.3	SSH1	SEC61A1/2	translocon subunit Sec61 homolog	transmembrane transport
C1039.01	-2.5	-3.5	TPO5, UGA4, HNM1	-	amino acid permease	transmembrane transport
meu29	-2	-2.5	-	SARAF	calcium transport regulatory factor	transmembrane transport
ctr5	-1.5	-2.5	CTR3	SLC31A1/2	copper transporter complex subunit	transmembrane transport
ryh1	-4	-4	YPT6	RAB6A/B/C	GTPase	Signaling
ste20	-2	-2.5	TSC11	RICTOR	Rictor homolog	Signaling
pal1	-3.5	-4	PAL1	-	membrane associated protein	cell wall organization or biogenesis
kin1	-3	-3.3	KIN1/2	PRKAA1/2	microtubule affinity-regulating kinase	actin cytoskeleton organization
hob3	-2.5	-3.5	RVS161	BIN3	BAR adaptor protein	actin cytoskeleton organization

Table S1 (Continued)

S. pombe	**10 μM**	**20 μM**	***S. cerevisiae***	***H. sapiens***	**Product**	**Representative GO slim**
imp2	-2.5	-3	HOF1	PSTPIP1/2	contractile ring protein	actin cytoskeleton organization
emc1	-2	-2	EMC1	EMC1	ER membrane protein complex subunit	protein folding
C323.04	-4	-4	YDR061W	-	mitochondrial ATPase	-
C27E2.11c	-1	-0.5	-	-	*Schizosaccharomyces* specific protein	-
C30D11.11	0	-2	IZH3	PAQR3	Haemolysin-III family protein	-
ypa1	0	-1.3	RRD1	-	protein phosphatase type 2A regulator, PTPA family	-

List of genes whose deletions exhibited sensitivity to FTY720 indicates the systemic/common name of the gene from *S. pombe* (the column labels; "*S. pombe*"),.the FTY720-sensitivities (the column labels; "10 μM", "20 μM"), orthologs (the column labels; "*S.cerevisiae*", "*Homo sapiens*"), a brief description of the function of each gene product. (the column labels; "Product"), and Representative GO slim FTY720-sensitivity expressed the average of the duplicate dilution-series spot test results. Ultimately, 39 mutants were scored as severely sensitive (the average of the scores on the 10 μM FTY720-containing YES plates were <0), 10 mutants were scored as mildly sensitive (the average of the scores on the 10 μM FTY720-containing YES plates were 0 and the average of the scores on the 20 μM FTY720-containing YES plates were <0)).

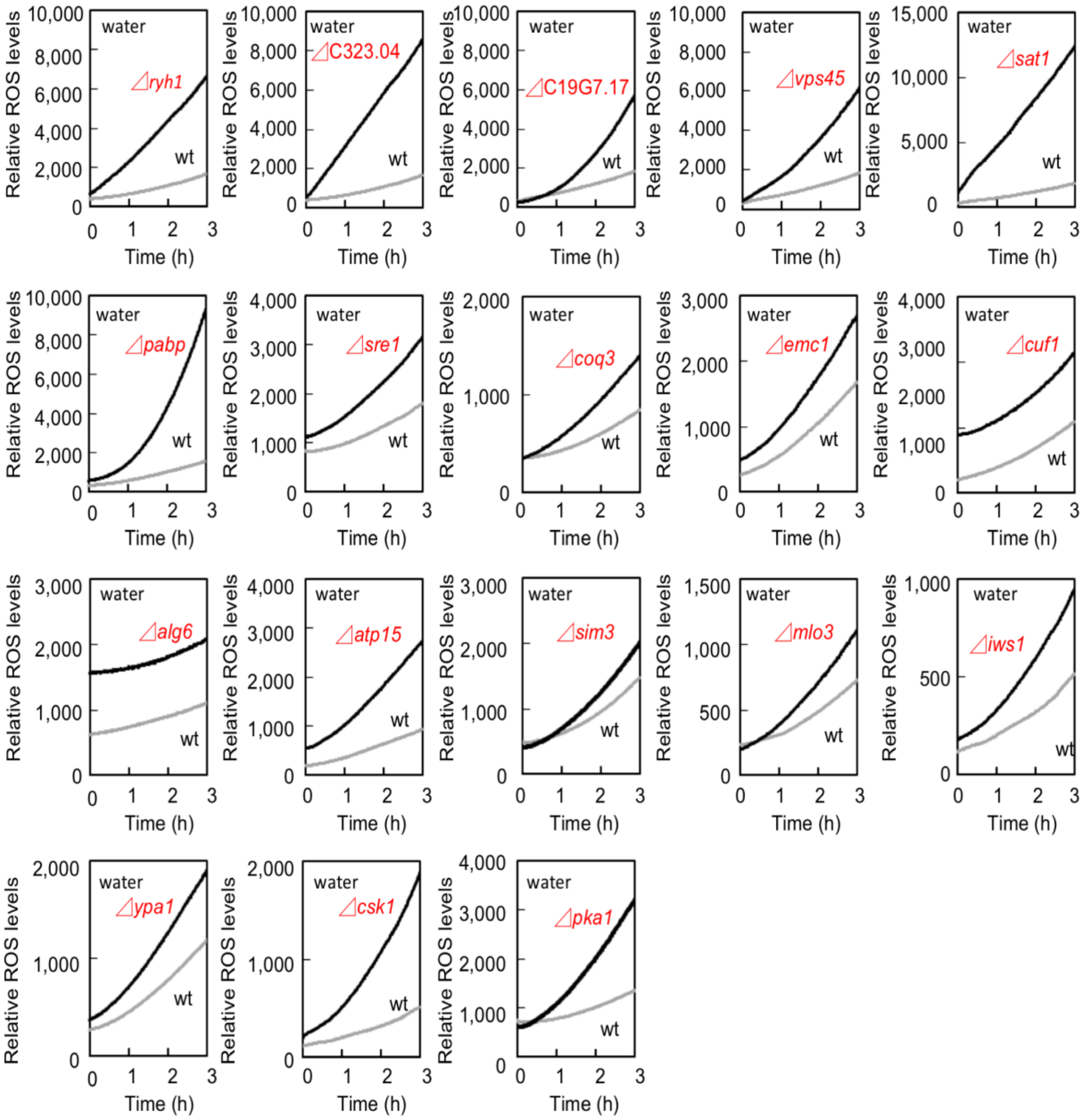

FIGURE S1: Select FTY720-sensitive mutants (Group1) from the screen were subjected to water, and ROS accumulation was monitored for 3 h.

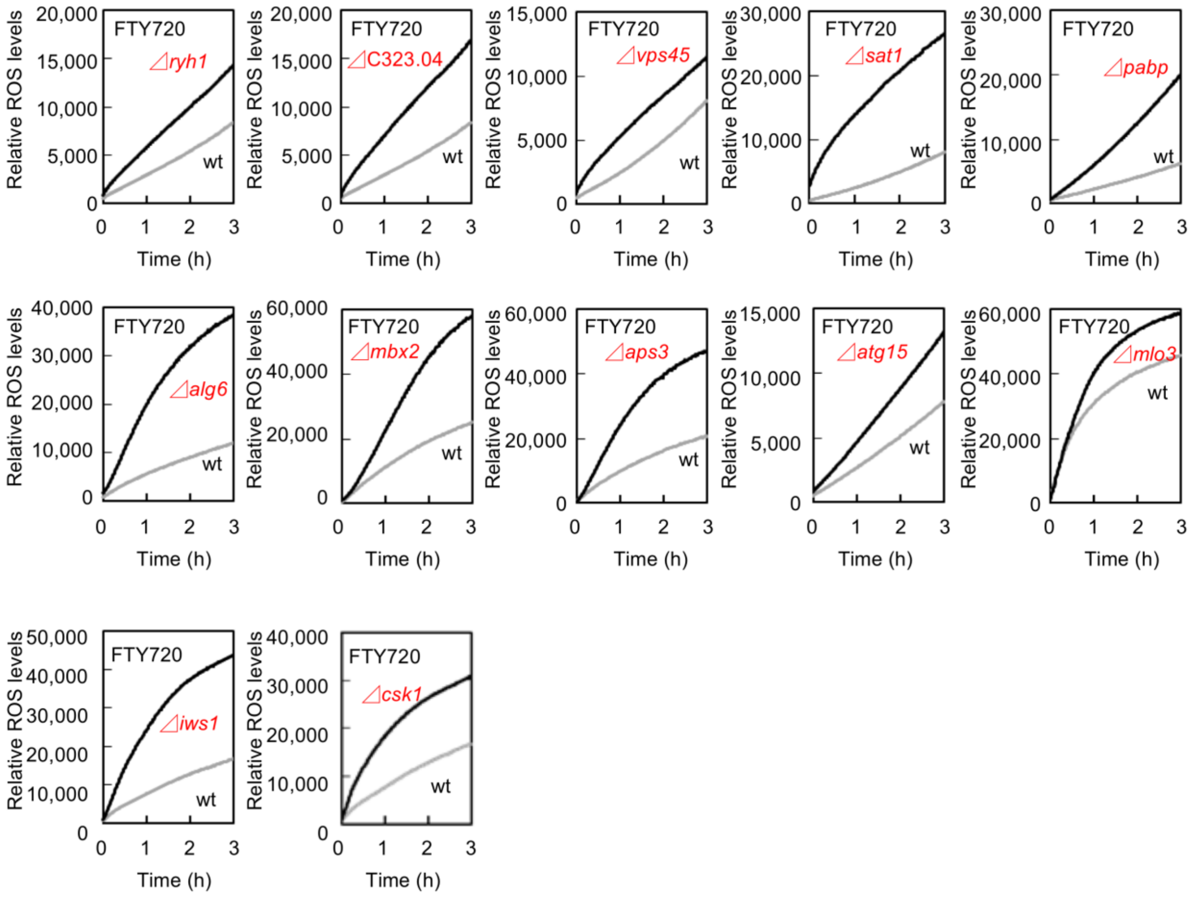

FIGURE S2: Select FTY720-sensitive mutants (Group1) from the screen were subjected to 30 μM FTY720, and ROS accumulation was monitored for 3 h.

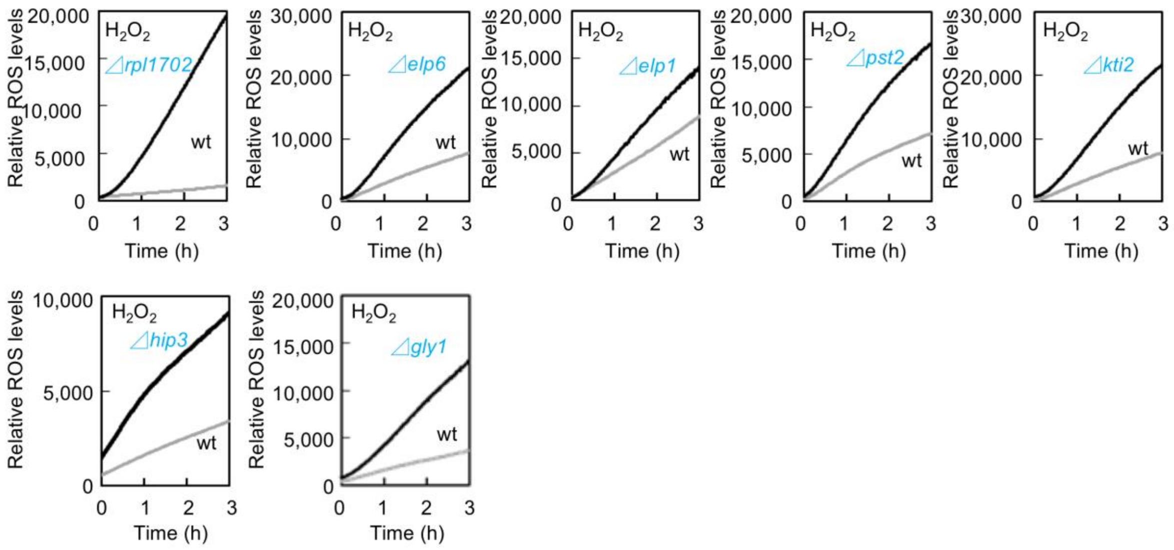

FIGURE S3: Select FTY720-sensitive mutants (Group2) from the screen were subjected to 2 mM H_2O_2, and ROS accumulation was monitored for 3 h.

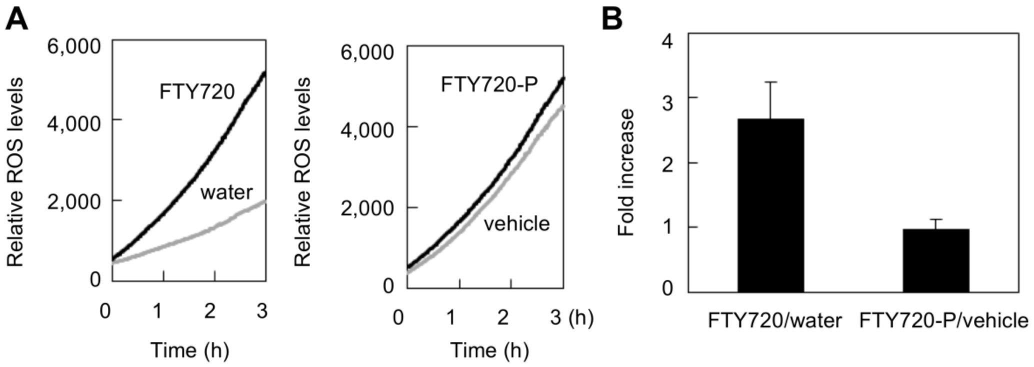

FIGURE S4: FTY720-P failed to stimulate ROS production (A) Effect of FTY720 and FTY720-P on ROS generation. Left panel: measurement of ROS production for 3 h in wt strain (h^- *leu1*) treated with vehicle (water alone) or 20 µM FTY720. Right panel: measurement of ROS production in wt strain (h^- *leu1*) treated with vehicle (80% ethanol containing 10 mM sodium hydroxide) or 20 µM FTY720-P. ROS accumulation was measured for 3 h (Materials and Methods). The data shown are representative of multiple experiments. (B) Histograms show the average of peak heights from three independent experiments shown in (A). Error bars, SD.
